# *Azadirachta indica* Attenuates Cisplatin-Induced Nephrotoxicity and Oxidative Stress

**DOI:** 10.1155/2014/647131

**Published:** 2014-08-03

**Authors:** Ahmed E. Abdel Moneim, Mohamed S. Othman, Ahmed M. Aref

**Affiliations:** ^1^Department of Biochemistry and Molecular Biology, Asturias Institute of Biotechnology, University of Oviedo, 33006 Oviedo, Spain; ^2^Department of Zoology and Entomology, Faculty of Science, Helwan University, Cairo 11795, Egypt; ^3^Department of Biochemistry and Molecular Biology, Faculty of Biotechnology, Modern Sciences and Arts University (MSA), Giza 12111, Egypt; ^4^Department of Biological Science, Faculty of Dentistry, Modern Sciences and Arts University (MSA), Giza 12111, Egypt

## Abstract

We investigated the effects of methanolic leaves extract of *Azadirachta indica* (MLEN, 500 mg/kg bwt) on cisplatin- (CP-) induced nephrotoxicity and oxidative stress in rats. CP (5 mg/kg bwt) was injected intraperitoneally and MLEN was given by gastric gavage for 5 days before or after CP injection. After 5 days of CP injection, CP-induced injury of the renal tissue was evidenced (i) as histopathological damage of the renal tissue, (ii) as increases in serum uric acid, urea, and creatinine, (iii) as increases in malondialdehyde (MDA) and nitric oxide (NO), (iv) as decreases in the level of glutathione and activities of superoxide dismutase, catalase, glutathione reductase, glutathione-S-transferase, and glutathione peroxidase, and (v) as increase in the expression of nuclear factor kappa B and apoptosis in kidney tissues. However, the oral administration of MLEN to CP-intoxicated rats for 5 days brought back MDA, NO production, and enzymatic and nonenzymatic antioxidants to near normalcy. Moreover, the histological observations evidenced that neem extract effectively rescues the kidney from CP-mediated oxidative damage. Furthermore, PCR results for caspase-3 and caspase-9 and Bax genes showed downregulation in MLEN treated groups. Therefore, *Azadirachta indica* can be considered a potential candidate for protection of nephrotoxicity induced by cisplatin.

## 1. Introduction

Cisplatin,* cis*-diamminedichloroplatinum (CP), with the molecular formula* cis*-[Pt(NH_3_)_2_Cl_2_], is one of the most remarkable platinum-based drugs used in “the war on cancer” [1–3]. CP and related platinum-based therapeutics are being used for the treatment of testicular, head and neck, ovarian, cervical, and non-small cell lung carcinoma and many other types of cancer. Its chief dose-limiting side effect is nephrotoxicity. A total of 20% of patients receiving high-dose CP have severe renal dysfunction. CP-DNA cross links cause cytotoxic lesions in tumours and other dividing cells. DNA damaging agents usually have less toxicity in nonproliferating cells, yet the quiescent proximal tubule cells are selectively damaged by CP which accumulates in the kidney to a greater degree than in other organs [4]. The mechanism for this renal cell injury has been the focus of intense investigation for many years. Recent studies suggest that inflammation, oxidative stress injury, and apoptosis probably explain part of this injury [5]. In animals, treated with CP for tumours, increased levels of trace elements, for example, iron, copper, and zinc, in addition to platinum, were observed in the kidneys and liver. This suggested that platinum toxicity is due to an overall accumulation of trace elements in these organs and is attributable to platinum alone [6].

CP causes generation of reactive oxygen species (ROS), such as superoxide anion and hydroxyl radical which deplete glutathione levels and inhibit the activity of antioxidant enzymes in renal tissue. ROS may produce cellular injury and necrosis via several mechanisms including peroxidation of membrane lipids, protein denaturation, and DNA damage [7].

Foods of plant origin with diverse medicinal properties have come under extensive study in light of their antioxidant, antimutagenic, and anticarcinogenic effects [8, 9]. In particular,* Azadirachta indica* (*A. indica*, neem) is one of the most promising medicinal plants, having a wide spectrum of biological activities, well known for its insecticidal properties. Several studies have been undertaken on the protective effects of neem [10–13]. Aqueous neem leaves extract was found to be well tolerated and dose up to 2.5 g/kg (single administration) or 1 g/kg when given for 28 days did not cause any mortality or histological changes in the kidney, liver, testis, or adrenals [14]. Every part of neem tree has been known to possess a wide range of pharmacological properties, especially as antibacterial, antifungal, and antiulcer [15].

Neem leaf consists of several valuable components and can be divided into two major classes: isoprenoids that include terpenoids containing limonoids and azadirone. The nonisoprenoids include amino acids, polysaccharides, sulphurous compounds, and polyphenolics, for example, quercetin, dihydrochalcone, coumarin, and tannins [16]. Water soluble extract of* A. indica* leaves was found to possess significant hypoglycemic, hypolipidemic, hepatoprotective, antifertility, and hypotensive activities [12]. In a study by Akinola et al. [17] they found that leaf extract of* A. indica* ameliorates hyperglycemia and diabetic nephropathy in rats.

The aim of the present study was to evaluate the possible protective effects of methanolic leaves extract of* Azadirachta indica* on CP-induced nephrotoxicity and oxidative stress.

## 2. Materials and Methods

### 2.1. Chemicals

*cis*-Diammine platinum (II) dichloride, nitro blue tetrazolium, N-(1-naphthyl) ethylenediamine, and Tris-HCl were purchased from Sigma (St. Louis, MO, USA). Thiobarbituric acid and trichloroacetic acid were purchased from Merck. All other chemicals and reagents used in this study were of analytical grade. Double-distilled water was used as the solvent.

### 2.2. Preparation of Neem Leaves Extract

The neem leaves were harvested from middle aged green trees from garden in Al Obour City, Cairo, on August 2011. The samples were identified in Botany Department, Faculty of Science, Helwan University. The leaves were cleaned and sun-dried for three days on hygienic cement floors until they became crispy but still retaining the greenish tint and after then they were powdered. The powder was used for the preparation of crude methanolic extract according to the procedure described by Manikandan et al. [18] with some modification. Air-dried powder (100 g) of* A. indica* leaves was extracted by percolation with 70% methanol alcohol and kept in refrigerator for 24 hours. Leaves extract of* A. indica* was concentrated under reduced pressure (bath temperature 45°C) and dried in a vacuum evaporator. The residue was dissolved in distilled water, filtered, and kept until being used in the experiment. The plant extract was subjected to screening for various phytochemicals. The quantities of phenolics and flavonoids were established in the MLEN (Table 1).

### 2.3. Animals and Experimental Design

Adult female albino Wistar rats weighing 150–170 g were obtained from The Holding Company for Biological Products and Vaccines (VACSERA, Cairo, Egypt). After an acclimatization period of one week, the animals were divided into four groups (7 rats per group) and housed in wire bottomed cages in a room under standard conditions of illumination with a 12-hour light-dark cycle at 25 ± 1°C. They were provided with water and a balanced diet* ad libitum*. All animals received care in compliance with the Egyptian rules for animal protection.

Group I (Con; control group) served as untreated control, Group II (CP group) received a single intraperitoneal injection of CP (5 mg/kg bwt) and was left for 5 days, Group III (N-CP group) received a daily oral administration of 500 mg/kg bwt neem leaves extract for 5 days before a single intraperitoneal injection of CP, and Group IV (CP-N group) received the same dose of the extract for 5 days following a single intraperitoneal injection of CP. MLEN group has received an oral administration of MLEN (500 mg/kg bwt) for 5 consecutive days via epigastric tube and its results were represented as supplementary data.

The level of the orally administered dosage of MLEN (500 mg/kg bwt) was based on the previous work of Ezz-Din et al. [12] and the dose of CP was selected on the basis of the previous studies [7, 12].

The animals of all groups were sacrificed by fast decapitation; blood samples were collected, allowed to stand for half an hour, and then centrifuged at 3000 rpm for 15 min at 4°C to separate serum which was stored at −20°C for different biochemical measurements. Right kidney was dissected out and fixed immediately in 10% neutral formaldehyde for histological and immunohistochemical studies. Left kidney was weighed and homogenized immediately to give 50% (w/v) homogenate in ice-cold medium containing 50 mM Tris-HCl, pH 7.4. The homogenate was centrifuged at 3000 rpm for 10 min at 4°C. The supernatant was used for various biochemical determinations.

### 2.4. Relative Kidney Weight

At the end of the experimental period, each rat was weighed. The left kidney was then removed and weighed. Finally, the relative kidney weight was calculated by dividing left kidney weight by body weight and then multiplying it by 100.

### 2.5. Biochemical Estimations

#### 2.5.1. Kidney Functions Tests

Serum creatinine, urea, and uric acid were determined by commercially available diagnostic kits (Diamond Diagnostics, Egypt) according to the manufacturer's instructions.

#### 2.5.2. Oxidative Stress Markers

Nitrite/nitrate (NO) and malondialdehyde (MDA) were assayed colorimetrically in kidney homogenates according to the methods of Green et al. [19] and Ohkawa et al. [20], respectively, where MDA was determined by using 1 mL of trichloroacetic acid 10% and 1 mL of thiobarbituric acid 0.67% which were then heated in a boiling water bath for 30 min. Thiobarbituric acid reactive substances (TBARS) were determined by the absorbance at 535 nm and expressed as MDA formed. Nitric oxide was determined where in acid medium and in the presence of nitrite the formed nitrous acid diazotised sulphanilamide is coupled with N-(1-naphthyl) ethylenediamine. The resulting azo dye had a bright reddish-purple color which can be measured at 540 nm.

In addition, the renal glutathione (GSH) was determined by the methods of Ellman [21]. This method is based on the reduction of Elman's reagent (5,5′-dithiobis (2-nitrobenzoic acid) “DTNB”) with GSH to produce a yellow compound. The reduced chromogen was directly proportional to GSH concentration and its absorbance can be measured at 405 nm.

#### 2.5.3. Enzymatic Antioxidant Status

The activities of renal antioxidant enzymes as catalase (CAT) were assayed by the method of Aebi [22]. As a result, CAT reacts with a known quantity of H_2_O_2_. The reaction is stopped exactly after one minute with catalase inhibitor. In the presence of peroxidase (HRP), the remaining H_2_O_2_ reacts with 3,5-dichloro-2-hydroxybenzene sulfonic acid (DHBS) and 4-aminophenazone (AAP) to form a chromophore with a color intensity that is inversely proportional to the amount of CAT in the original sample. The resulting chromophore had a bright rose color which can be measured at 510 nm. Renal superoxide dismutase (SOD) activity was assayed by the method of Nishikimi et al. [23]. This assay relies on the ability of the enzyme to inhibit the phenazine methosulphate-mediated reduction of nitroblue tetrazolium dye. Glutathione-S-transferase (GST) activity in the kidney was assayed by the method of  Habig et al. [24]. The total GST activity was assayed by measuring the conjugation of 1-chloro-2,4-dinitrobenzene (CDNB) with reduced glutathione. The conjugation is accompanied by an increase in absorbance at 340 nm. The rate of increase is directly proportional to the GST activity in the sample. Renal glutathione peroxidase (GPx) activity was measured by the method of Paglia and Valentine [25] and the assay was an indirect measurement of the activity of GPx. Oxidized glutathione, produced upon reduction of organic peroxide by GPx, was recycled to its reduced state by the enzyme glutathione reductase (GR). The reaction was initiated by the addition of hydrogen peroxide, and the oxidation of NADPH to NADP^+^ is accompanied by a decrease in absorbance at 340 nm. Finally, glutathione reductase activity of the kidney was assayed by the method of Factor et al. [26]. GR catalyzes the reduction of glutathione in the presence of NADPH, which is oxidized to NADPH^+^. As a result, the decrease in absorbance at 340 nm was measured.

#### 2.5.4. Real Time PCR

Total RNA was isolated from the kidney tissue using an RNeasy Plus Minikit (Qiagen, Valencia, CA). One microgram total RNA and random primers were used for cDNA synthesis using the RevertAid H Minus Reverse Transcriptase (Fermentas, Thermo Fisher Scientific Inc., Canada). For real time PCR analysis, the cDNA samples are run in triplicate and *β*-actin is used as reference gene. Real time PCR reactions were performed using Power SYBR Green (Life Technologies, CA) and were conducted on the Applied Biosystems 7500 Instrument. The typical thermal profile is 95°C for 3 min, followed by 40 cycles of 95°C for 15 s and 56°C for 30 s. After PCR amplification, the ΔCt is calculated by subtraction of the *β*-actin Ct from each sample Ct. The PCR primers for caspase-3, caspase-9, and Bax genes were synthesized by Jena Bioscience GmbH (Jena, Germany). Primers were designed using Primer-Blast program from NCBI. The PCR primer sequences are BLAST searched to insure specificity to this particular gene. The primer sets used were rat caspase-3 (forward: 5′-GCATGATCCGCGACGTGGAA-3′, reverse: 5′-AGATCCATGCCGTTGGCCAG-3′), rat caspase-9 (forward: 5′-ATGCAGGTCCCTGTCATG-3′, reverse: GCTTGAGGTGGTTGTGGA-3′), rat Bax (forward: 5′-AGATCACATTCACGGTGCTG-3′, reverse: 5′-CTTCAGAGGCAGGAAACAGG-3′), and *β*-actin (forward: 5′-AGAGGGAAATCGTGCGTGAC-3′, reverse: 5′-CAATAGTGATGACCTGGCCGT-3′).

#### 2.5.5. Histopathological Examination

Tissue samples were fixed in 10% neutral formalin for 24 h and paraffin blocks were obtained and routinely processed for light microscopy. Slices of 4-5 *μ*m were obtained from the prepared blocks and stained with hematoxylin-eosin. The preparations obtained were visualized under a microscope (Eclipse E200-LED, Nikon, Tokyo, Japan).

#### 2.5.6. Immunohistochemistry for Detection of Nuclear Factor Kappa B (NF-*κ*B)

Detection of NF-*κ*B was performed according to Pedrycz and Czerny [27]. Kidney sections (4-5 *μ*m) were deparaffinized and then boiled in Declere (Cell Marque, Hot Springs, AR, USA) to unmask antigen sites; the endogenous activity of peroxidase was quenched with 0.03% H_2_O_2_ in absolute methanol. Kidney sections were incubated overnight at 4°C with a 1 : 200 dilution of mouse anti-NF-*κ*B antibodies (Santa Cruz Biotechnology) in phosphate buffered saline (PBS). Following removal of the primary antibodies and repetitive rinsing with PBS, slides were incubated with a 1 : 500 dilution of biotinylated anti-mouse secondary antibody (Santa Cruz Biotechnology). Bound antibodies were detected with avidin biotinylated peroxidase complex ABC-kit Vectastain (Vector Laboratories, Burlingame, CA, USA) and diaminobenzidine substrate (Sigma Chemical Co.). After appropriate washing in PBS, slides were counterstained with hematoxylin (Sigma Chemical Co.) and mounted with DPX. The number of positive cells was scored using five categories: <5%, negative (category 1); 5–25% (category 2); 25–50% (category 3); 50–75% (category 4); 75–100% (category 5). Next, the staining intensity was graded as very week, week, medium, or dark. All sections were incubated under the same conditions with the same concentration of antibodies and at the same time, so the immunostaining was comparable among the different experimental groups.

#### 2.5.7. Detection of Apoptosis by Propidium Iodide Staining

Kidney sections were incubated with 4 mg/mL propidium iodide and 100 mg/mL RNase (Sigma Chemical Co., DNase-free) in PBS for 60 minutes at 37°C. The slides were washed in PBS, mounted, and examined with a Zeiss fluorescence microscope. The number of PI-positive cells in the kidney was counted in each section. Data are represented as findings per section.

#### 2.5.8. Statistical Analysis

Differences between obtained values (mean ± SEM) were carried out by one-way analysis of variance (ANOVA) followed by the Duncan test. A *P* value of 0.05 or less was taken as a criterion for a statistically significant difference.

## 3. Results

**Figure 1:**
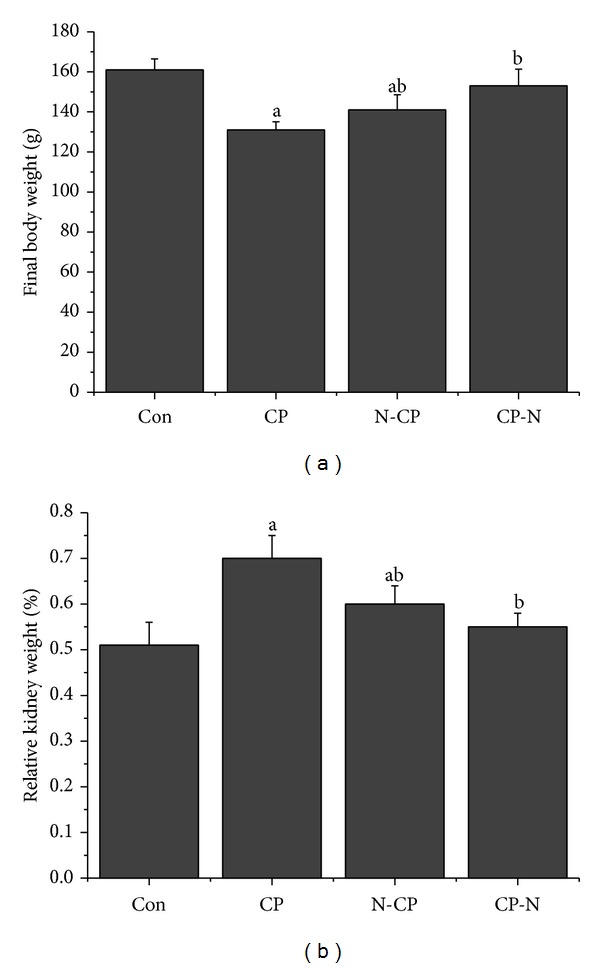
Effects of methanolic leaves extract of neem on body weight and relative kidney weight of rats treated with CP. Values are mean ± SEM (*n* = 7). ^a^ *P* < 0.05, significant change with respect to control; ^b^ *P* < 0.05, significant change with respect to CP for Duncan's post hoc test.

Comparing the final body weights of rats in different groups, a significant weight loss (*P* < 0.05) was detected in CP-treated rats. Conversely, a significant weight gain was recorded in the CP-N and N-CP treated rats when compared to the CP-treated rats. In CP-N treated rats group, a restoration of the body weight was observed compared to the CP-treated rats group. Moreover, a significant difference of relative kidney weight of the CP-treated and N-CP treated rats was noticed versus that of the control rats at *P* < 0.05. The relative kidney weights revealed significant difference (*P* < 0.05) in both CP-N and N-CP treated rats when compared to the CP-treated rats (Figure 1).

The levels of uric acid, urea, and creatinine were significantly elevated (*P* < 0.05) in the blood serum of rats of Group II by approximately 69.6%, 96.3%, and 50.9%, respectively (Table 2). However, a significant deference in the levels of the parameters mentioned above was recorded in CP-N and N-CP treated groups when compared to the CP-treated rats. Additionally, MLEN alone did not exhibit any effect on urea and creatinine levels after 5 days of treatment in the rats; moreover, the serum level of uric acid in MLEN-treated rats was increased significantly (Supplementary data, Table S1, in Supplementary Material available online at http://dx.doi.org/10.1155/2014/647131).

**Figure 2:**
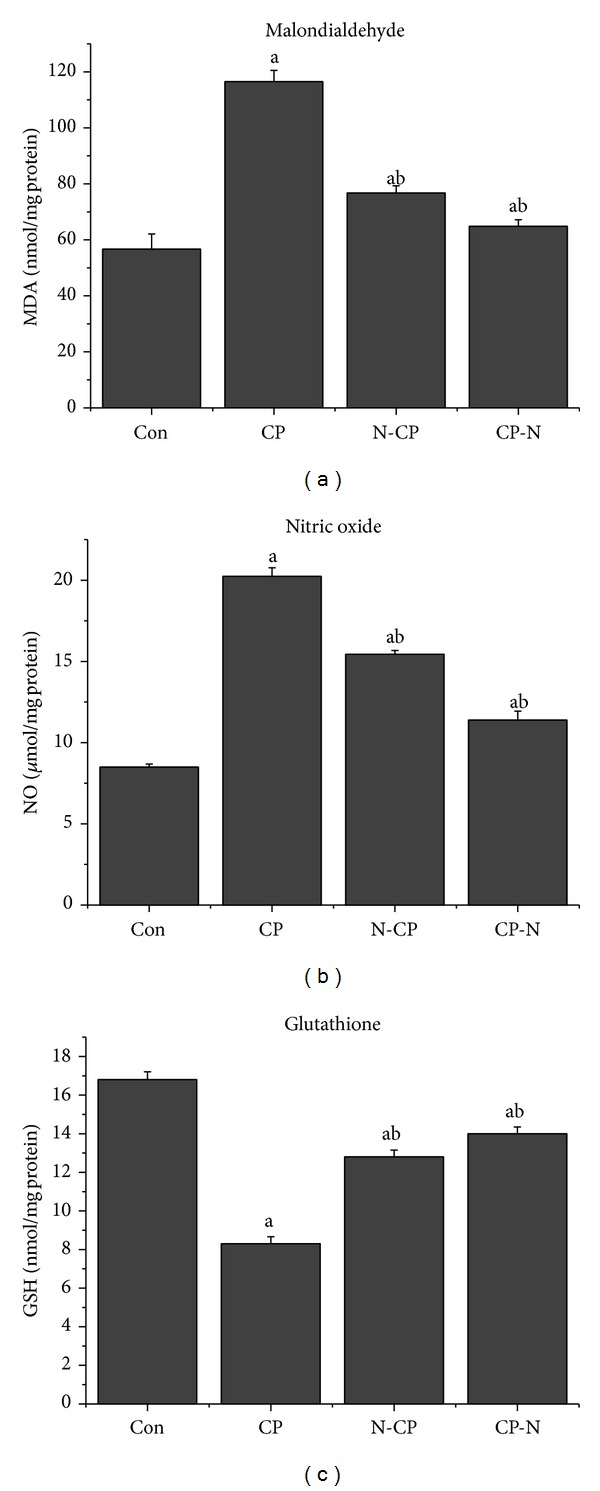
Effects of methanolic leaves extract of neem on malondialdehyde, nitric oxide, and glutathione levels in kidney of rats treated with CP. Values are mean ± SEM (*n* = 7). ^a^ *P* < 0.05, significant change with respect to control; ^b^ *P* < 0.05, significant change with respect to CP for Duncan's post hoc test.

Oxidative stress has been regarded as one of the underlying mechanisms of CP-induced acute organ injury [28]. We evaluated whether MLEN (500 mg/kg) could modulate CP-induced renal oxidative stress by measuring MDA and NO levels. CP-induced renal oxidant stress was evident by significant increase (*P* < 0.05) in MDA and NO levels and by a significant decrease (*P* < 0.05) in GSH content in the kidney tissues of animals that received CP alone compared with the control group. These changes in MDA, NO, and GSH levels were attenuated by treatment with MLEN as shown in Figure 2. Administration of MLEN alone for 5 days increased the renal NO concentration as compared with the control rats (Supplementary data, Table S1).

**Figure 3:**
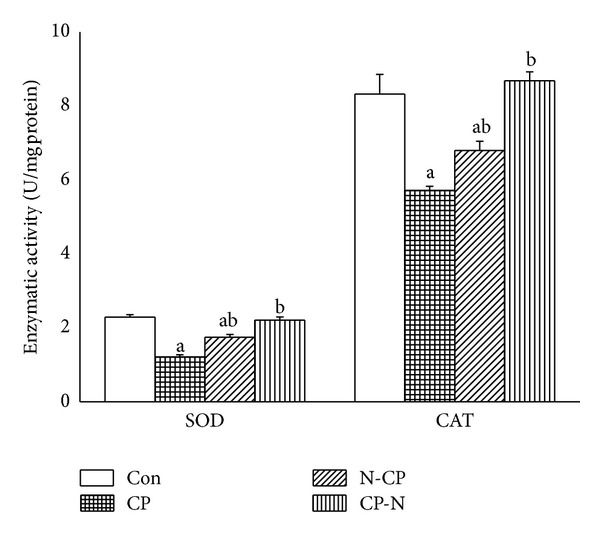
Effects of methanolic leaves extract of neem on superoxide dismutase and catalase activities in kidney of rats treated with CP. Values are mean ± SEM (*n* = 7). ^a^ *P* < 0.05, significant change with respect to control; ^b^ *P* < 0.05, significant change with respect to CP for Duncan's post hoc test.

Cisplatin intoxication resulted in significant decrease (*P* < 0.05) in renal antioxidant enzyme activities like SOD, CAT, GPx, GR, and GST in CP-treated rats compared with the control group. The presence of MLEN before or after CP injection normalized the activities of antioxidant enzymes to nearly the normal values of control (Table 3 and Figure 3). MLEN treatment* per se* did not alter the activities of SOD, CAT, and GST; however, MLEN at 500 mg/kg produced a significant (*P* < 0.05) increase in GR and GPx activities (Supplementary data, Table S1).

**Figure 4:**
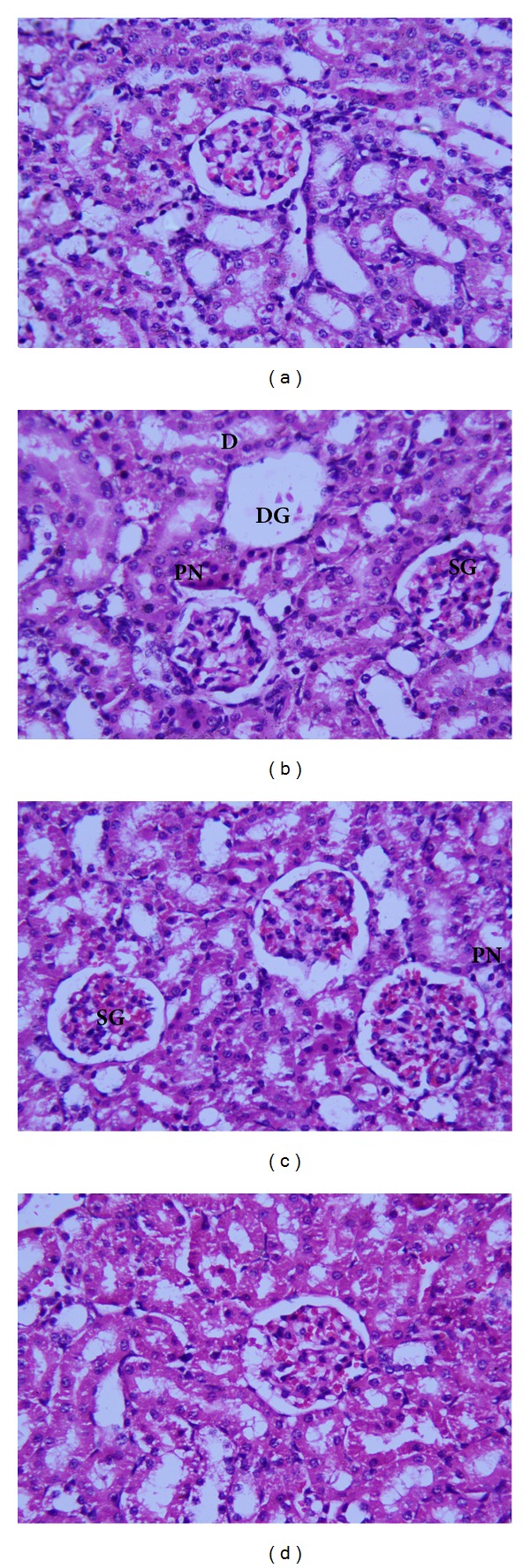
H&E staining showing histopathological damage induced by CP on the kidney of rats. (a) Control kidney section with normal renal corpuscle and renal tubules. (b) Rats treated with cisplatin with congested shrank and completely degenerated glomeruli, debris in the lumen of some renal tubules, and pyknotic nuclei in renal tubules. ((c) and (d)) Treatment with methanolic leaves extract of neem before or after administration of CP showing normal renal corpuscle and renal tubule more or less like normal structure (X 400). SG: shrank glomeruli, DG: degenerated glomeruli; D: debris; PN: pyknotic nuclei.

The renal cortex of control rats revealed a normal corpuscular and tubular histological structure (Figure 4(a)). In CP-treated rats, degenerative changes were noticeably observed in renal tissues. These changes were in the form of luminal dilatation with excessive cast accumulation; the renal corpuscles showed dilated capsular space with condensed and even degenerated glomerulus. Inflammatory cells infiltration and vascular congestion were noticed within the renal cortex and pyknotic nuclei were present in renal tubules (Figure 4(b)). The kidney of rats treated with MLEN to CP (Groups III and IV) revealed less histological damage in renal corpuscles and renal tubules. Mild tubular degeneration with luminal dilatation and inflammatory cell infiltration were seen within the renal cortex (Figures 4(c) and 4(d), resp.).

**Figure 5:**
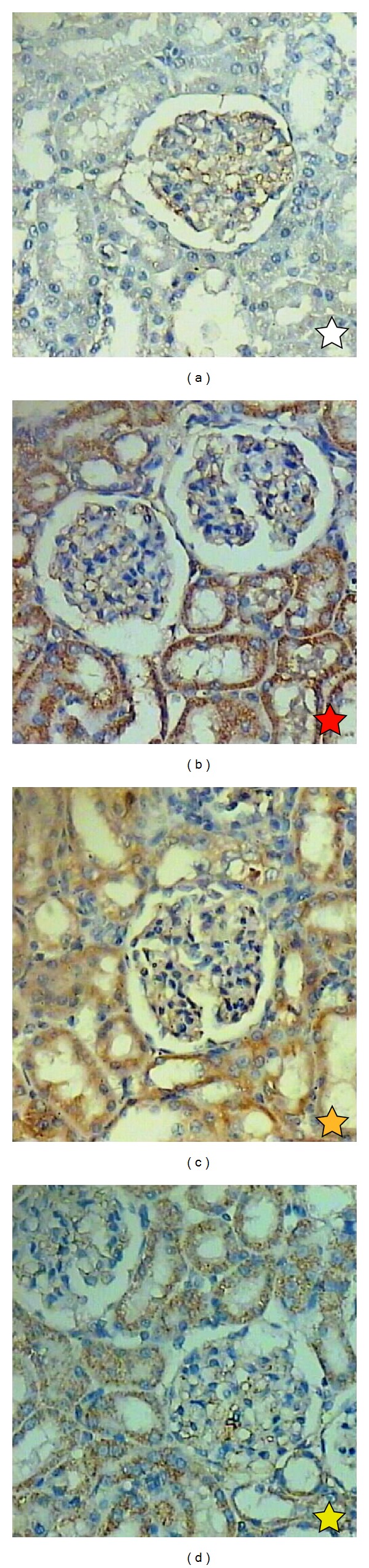
NF-*κ*B expression changes in the kidney of rats. (a) Normal kidney showing negative expression for NF-*κ*B. (b) Treated kidney with cisplatin showing positive expression for NF-*κ*B. (c) Kidney of rats treated with methanolic leaves extract of neem after 5 days of cisplatin injection showing mild positive expression for NF-*κ*B. (d) Treated kidney with cisplatin after methanolic leaves extract of neem showing moderate expression for NF-*κ*B (400X). The staining intensity was graded as white star (category 1); blue star (category 2); yellow star (category 3); orange star (category 4); red star (category 5).

NF-*κ*B plays a key role in the inflammation process during nephrotoxicity. As shown in Figure 5 the immunostaining activity of NF-*κ*B was markedly increased in renal tubular cells of CP-treated rats than the control group. Correspondingly, this increase in immunostaining activity of NF-*κ*B was decreased significantly by the treatment with MLEN. In this case, the numbers of NF-*κ*B immunostaining cells were decreased (Supplementary data, Table S2).

**Figure 6:**
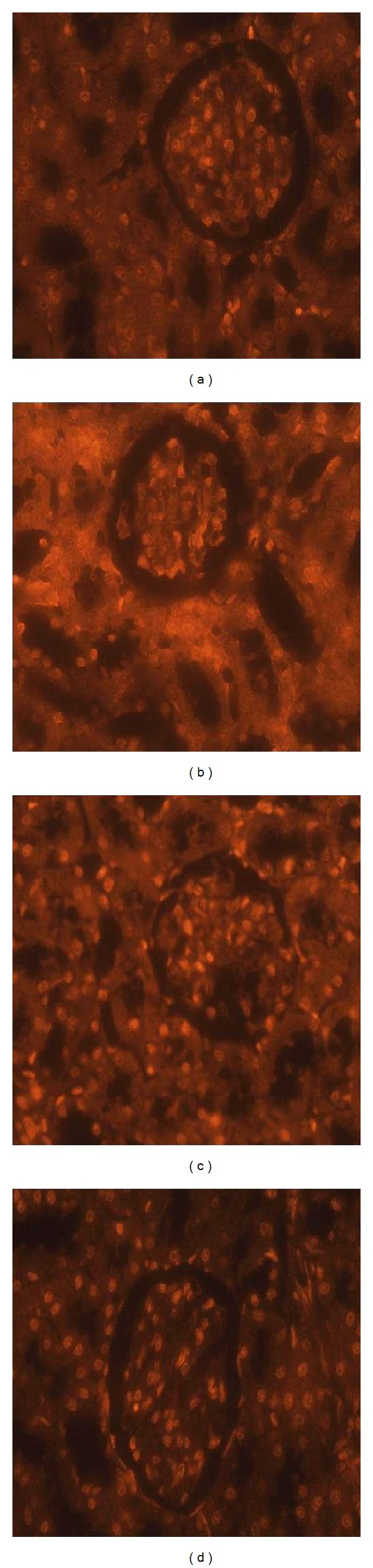
Morphological changes visualized under fluorescence microscope with PI staining in the kidney of rats. (a) Normal kidney. (b) Treated kidney with cisplatin. (c) Kidney of rats treated with methanolic leaves extract of neem after 5 days of cisplatin injection. (d) Treated kidney with cisplatin after methanolic leaves extract of neem. The bright cells indicate the cells undergoing apoptosis (400X).

Renal tubular apoptosis has been suggested as a mechanism of CP-induced acute kidney injury [29]. We further examined MLEN ability to reduce CP-mediated apoptosis in the kidney by PI staining. Increased PI staining cells were observed in CP-treated rats after 5 days of CP injection (Figure 6). Treatment with MLEN before or after CP injection substantially reduced the number of PI staining cells, which implied that the apoptotic cascade might play a key role in MLEN renoprotective effect. Quantitative analyses of PI-positive cells in the different treated groups are shown in Supplementary data, Table S2. PI-positive cells of control kidney were 13 ± 2 cells/section. The number of PI-positive cells was significantly increased in the CP-treated rats (164 ± 12 cells/section). The treatment with MLEN decreased this number significantly when compared with the CP-treated group, 51 ± 8 cells/section in the N-CP and 27 ± 9 cells/section in the CP-N.

**Figure 7:**
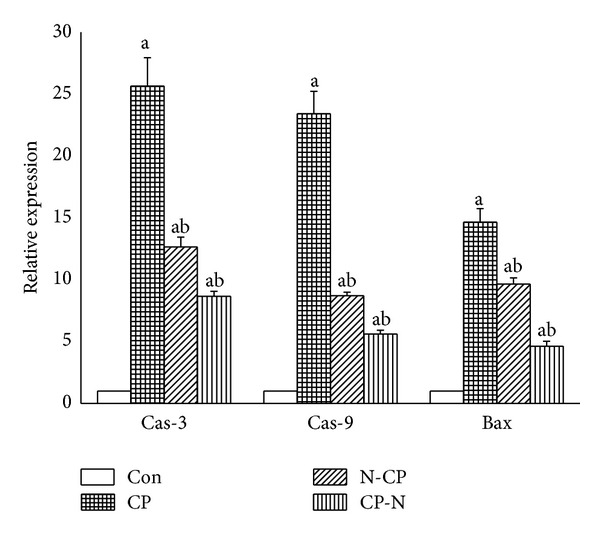
Relative quantification using RT-qPCR of mRNA expression of caspase-3 and caspase-9 and Bax genes in kidney of rats treated with cisplatin and methanolic leaves extract of neem.

In the reverse transcription RT-PCR analysis of apoptosis-related genes, expression of proapoptotic Bax, caspase-3, and caspase-9 genes increased significantly in kidneys of CP-injected rats compared to those of untreated control rats. Treatment with MLEN reversed the increased Bax, caspase-3, and caspase-9 mRNA expressions in kidneys of CP-injected rats (Figure 7).

## 4. Discussion

CP is one of the most widely used cytotoxic therapeutic agents for the treatment of different cancers including testicular, germ cell, head and neck, bladder, and lung cancers. It is an alkylating agent which at effective higher doses causes many adverse effects such as neurotoxicity, nephrotoxicity, and genotoxicity [12, 30]. CP-induced nephrotoxicity can result in severe renal tubular injury leading to acute renal failure [31]. Nephrotoxicity by CP occurs in a dose-dependent manner, is cumulative, and occurs to varying degrees in 25–35% of patients receiving a single dose of CP [32]. The CP concentration in proximal tubular epithelial cells is about 5 times the serum concentration. The disproportionate accumulation of CP in kidney tissue contributes to CP-induced nephrotoxicity. In a study by Yao et al. [33] they suggested that inflammation, oxidative stress injury, and apoptosis probably explain part of this injury. Nephrotoxicity has been a serious problem in CP treatment and combined chemotherapy using CP and plant extracts can reduce the side effects and enhance the antitumour efficacy [12, 34, 35].

Our results showed that administration of CP (5 mg/kg bwt) to rats caused a reduction in glomerular filtration rate, which is correlated with increased serum creatinine, urea, and uric acid. These results are in accordance with previous reports of Naziroğlu et al. [36], Ateşşahín et al. [37], and Naqshbandi et al. [38] who reported that the alterations induced by CP in the kidney functions were characterized by signs of injury, such as increase of creatinine and urea levels in plasma. However, the administration of MLEN before or after CP treatment significantly improved the kidney function. Moreover, our histological findings showed that treatments of MLEN with CP dramatically improved the CP nephrotoxicity and lead to less histological damage in renal corpuscles and renal tubules. These results are in agreement with Ezz-Din et al. [12].

Among many markers of oxidative stress, renal MDA and NO were reported to be increased following injection of CP. As shown in Figure 2 there were significant increases (*P* < 0.05) in MDA and NO levels in the kidney of animals that received CP alone compared with the control group. These results were in agreement with Bae and his colleagues [39], who demonstrated an increase in lipid peroxidation and inducible nitric oxide synthase (iNOS) mRNA levels in kidney 4 days after CP injection. iNOS is able to produce large amounts of nitric oxide that, under oxidative stress conditions, can react with superoxide anion (O_2_^•−^) to form nitrate (ONOO^−^), an oxidant species able to modify a great number of biomolecules such as amino acids, proteins, enzymes, and cofactors and involved in CP-induced nephrotoxicity. Moreover, MLEN can attenuate MDA and NO increments in kidney tissue.

CP initially triggers oxidative stress in the kidney, which is followed by a secondary wave of ROS/RNS (reactive nitrogen species) generation and an intense inflammatory response. ROS directly acts on cell components, including lipids, proteins, and DNA and destroys their structure. We demonstrated significant decreases (*P* < 0.05) in GSH level and SOD, CAT, GR, GST, and GPx activities in kidney of CP-treated rats as compared with the control group. In this study, we demonstrated that MLEN could restore renal injury induced by CP treatment and confirmed the important role of MLEN antioxidant property against CP-induced nephrotoxicity, in particular, via enhancing the antioxidant defence system.

In the past, studies on the pathogenesis of CP nephrotoxicity mainly focused on the direct toxicity such as oxidative stress. The administration of antioxidants has been shown to ameliorate CP-induced toxicity in rats [28]. CP can cause an increase in lipid peroxide and nitric oxide levels and a decrease in the activity of antioxidant defence enzymes that protect from oxidative stress in kidneys. This could explain the potent ability of MLEN to diminish MDA and NO levels against CP treatment. Consistently, Bandyopadhyay et al. [16] reported that some constituents of* A. indica* such as flavonoids (quercetin) have antioxidant activity so this plant may inhibit lipid peroxidation by scavenging free radicals and increasing intracellular concentration of glutathione. Moreover, the decline of SOD, CAT, GPx, GST, and GR activities as well as decrement of GSH content was exhibited after CP injection, resulting in the reduced ability of the kidney to scavenge toxic H_2_O_2_, O_2_^−^, and lipid peroxides. In our study, MLEN was able to restore SOD, CAT, GPx, GST, and GR activities in kidney tissues.

The central role of cytokines activity and inflammation in CP nephrotoxicity has been focused on recently [29]. Among them, NF-*κ*B has been reported to coordinate the activation of a large network of chemokines and cytokines in the kidney following CP injection. At the same time, NF-*κ*B could be an important molecule that possibly couples ROS to regulate CP nephrotoxicity, further exacerbating renal tissue damage. ROS can also activate NF-*κ*B that leads to increased expression of proinflammatory mediators which could intensify the cytotoxic effects of CP [28]. Moreover, antioxidants can attenuate ROS generation and NF-*κ*B activation and thus protect the renal cells against CP injury [29]. In our study, pre- or posttreatment with MLEN significantly decreased the expression of NF-*κ*B. These results were in agreement with Manikandan et al. [18], who concluded that neem leaf extract can inhibit NF-*κ*B expression.

We herein showed that Bax, caspase-3, and caspase-9 are critical for CP-induced apoptosis of kidney cells. Previous studies have reported association between caspase family members and cisplatin-induced apoptosis. CP may cause mitochondrial release of cytochrome c and caspase-9 and caspase-3 activation [40]. To examine the role of caspase in cisplatin-mediated apoptosis, we investigated the expression levels of caspase-9 and caspase-3. Our results revealed that caspase-9 and caspase-3 expression was induced in CP-treated kidney. In addition, Bcl-2 family members are also involved in CP-induced apoptosis. It has been observed that CP-induced apoptosis in both sensitive and resistant ovarian cancer cells is associated with an increased level of Bax and Bak proteins [40]. The present study showed that Bax was upregulated in CP-treated rats. N-CP or CP-N changed the expressions of the apoptotic genes. In a previous study, it has been shown that neem extract can be effective in CP-induced hepatotoxicity associated with apoptosis [28]. The present study here has demonstrated that MLEN administration significantly reduced CP-induced apoptosis in the kidney cells. These results suggest that one of the protective mechanisms of MLEN against CP nephrotoxicity is related to reduction of apoptosis.

These renoprotective actions of MLEN against CP nephrotoxicity may be due to its unique composition, where the leaves extract of neem is rich in flavonoids (rutin and quercetin, flavonoglycosides, polyphenolics, tannins, and so forth). Flavonoids in neem have been reported to possess both antioxidant and anti-inflammatory activities via scavenging free radicals and inhibition of  lipid peroxidation. In addition, neem leaves are rich in polyphenolics, which are known for their potent antioxidant and free radical scavenging properties [41].

In conclusion, the present study demonstrated that MLEN provided a significant protective effect against cisplatin-induced nephrotoxicity when administered following cisplatin injection and the mechanism of nephroprotection by MLEN could be due to the antioxidant, anti-inflammatory, and free radical scavenging activities of the neem.

## Supplementary Material

Supplementary Material 1: Effects of methanolic leaves extract of neem on different parameters of rats.
Supplementary Material 2: Quantitative analysis of NF-*κ*B immunoreactivity and PI cells in the kidney of rats on different treated groups .

## Figures and Tables

**Table 1:** Total phenolic and flavonoid contents of methanolic leaves extract of neem.

Phytochemical constituent	Total phenolics^a^	Total flavonoids^b^
Methanolic leaves extract of neem	83.71 ± 5.32	62.85 ± 4.66

^a^Total phenolics are expressed as *μ*g/mg gallic acid equivalent of phenolics/mg extract. ^b^Total flavonoids are expressed as *μ*g/mg quercetin equivalents of flavonoids/mg extract. Data are represented as mean ± SEM of two independent experiments, each performed in duplicate.

**Table 2:** Protective effects of methanolic leaves extract of neem on cisplatin-induced alternation in kidney function parameters of rats.

Groups	Uric acid (mg/dL)	Urea (mg/dL)	Creatinine (mg/dL)
Con	55.76 ± 2.63	2.97 ± 0.15	0.55 ± 0.04
CP	89.86 ± 4.69^a^	5.83 ± 0.29^a^	0.83 ± 0.04^a^
N-CP	71.64 ± 3.17^ab^	4.30 ± 0.26^ab^	0.71 ± 0.03^ab^
CP-N	65.62 ± 2.39^ab^	3.47 ± 0.18^ab^	0.64 ± 0.02^ab^

Values are mean ± SEM (*n* = 7).

^a^ *P* < 0.05, significant change with respect to control; ^b^ *P* < 0.05, significant change with respect to CP for Duncan's post hoc test.

**Table 3:** Protective effects of methanolic leaves extract of neem on cisplatin-induced alternation in enzymatic antioxidant molecules of rats.

Groups	GR (*μ*mol/hr/mg protein)	GST (*μ*mol/hr/mg protein)	GPx (U/mg protein)
Con	12.43 ± 1.05	0.07 ± 0.003	64.37 ± 3.36
CP	6.31 ± 0.78^a^	0.02 ± 0.003^a^	35.19 ± 2.03^a^
N-CP	15.76 ± 1.36^ab^	0.07 ± 0.003^b^	61.70 ± 4.87^b^
CP-N	22.10 ± 1.53^ab^	0.09 ± 0.004^ab^	75.49 ± 5.11^ab^

Values are mean ± SEM (*n* = 7).

^a^ *P* < 0.05, significant change with respect to control; ^b^ *P* < 0.05, significant change with respect to CP for Duncan's post hoc test.
