# Supplementary material for: Azadirachta indica Attenuates Cisplatin-Induced Nephrotoxicity and Oxidative Stress
Source: Biomed Res Int. 2014 Aug 3;2014:647131. doi: 10.1155/2014/647131 (PMC4137610; doi:10.1155/2014/647131)
Supplement: Supplementary file 1 — Supplementary Material 1: Effects of methanolic leaves extract of neem on different parameters of rats. Supplementary Material 2: Quantitative analysis of NF-κB immunoreactivity and PI cells in the kidney of rats on different treated groups . [file 647131.f1.pdf]

Table S1. Effects of methanolic leaves extract of neem on different parameters of rats.

	Groups	Uric acid (mg/dL)		Urea (mg/dL)		Creatinine (mg/dL)
**Kidney function**	**Control**	55.76±2.63		2.97±0.15		0.55±0.04
	**MLEN**	63.55±1.11*		2.77±0.03		0.51±0.01
		**MDA** (nmol/mg protein)		**NO** (μmol/mg protein)		**GSH** (nmol/mg protein)
**Oxidant markers**	**Control**	50.66±2.43		8.15±0.34		16.72±0.41
	**MLEN**	56.49±2.29		10.76±0.64*		17.25±0.47
		**SOD** (U/mg protein)	**CAT** (U/mg protein)	**GR** (μmol/hr/mg protein)	**GST** (μmol/hr/mg protein)	**GPx** (U/mg protein)
**Antioxidant enzymes**	**Control**	2.52±0.16	9.16±0.58	12.43±1.05	0.07±0.003	64.37±3.36
	**MLEN**	2.31±0.22	9.71±0.36	16.76±1.12*	0.07±0.002	75.32±2.74*

Values are means ± SEM (n=7)

*p<0.05, significant change with respect to **Control**.

Table S2. Quantitative analysis of NF-κB immunoreactivity and PI cells in the kidney of rats on different treated groups.

Units	Control	CP	N-CP	PC-N
**Average % NF-κB positive cells in the kidney**	2.4±0.41	89.6±3.54	65.3±4.78[ab]	28.5±2.82[ab]
**Number of PI positive cells/section**	13±2	164±21	51±8[ab]	27±9[ab]

[a]*p*<0.05, significant change with respect to **Control**; [b]*p*<0.05, significant change with respect to **CP** for Duncan's post hoc test.
